# {2,6-Bis[(2,6-diphenyl­phosphan­yl)­oxy]-4-fluoro­phenyl-κ^3^ *P*,*C* ^1^,*P*′}(6-methyl-2,2,4-trioxo-3,4-dihydro-1,2,3-oxathia­zin-3-ido-κ*N*)palladium(II)

**DOI:** 10.1107/S1600536811002911

**Published:** 2011-01-29

**Authors:** Benjamin F. Wicker, Rachel Seaman, Norris W. Hoffman, James H. Davis, Richard E. Sykora

**Affiliations:** a Department of Chemistry, University of South Alabama, Mobile AL 36688-0002, USA

## Abstract

The title acesulfamate complex, [Pd(C_30_H_22_FO_2_P_2_)(C_4_H_4_NO_4_S)], contains a four-coordinate Pd(II) ion with the expected, although relatively distorted, square-planar geometry where the four *L*—Pd—*L* angles range from 79.58 (8) to 102.47 (7)°. The acesulfamate ligand is N-bound to Pd [Pd—N = 2.127 (2) Å] with a dihedral angle of 76.35 (6)° relative to the square plane. Relatively long phen­yl–acesulfamate C—H⋯O and phen­yl–fluorine C—H⋯F inter­actions consolidate the crystal packing.

## Related literature

For the low toxicity of acesulfamate, see: Lipinski (2003) ▶. For examples of different modes of acesulfamate bonding to transition metals, see: Bulut *et al.* (2005 ▶); Cavicchioli *et al.* (2010 ▶); Şahin *et al.* (2009 ▶, 2010 ▶); Dege *et al.* (2006 ▶, 2007 ▶); Beck *et al.* (1985 ▶); İçbudak *et al.* (2005 ▶). For applications of ^19^F-NMR reporter moieties in monitoring ligand-substitution equilibria, see: Hoffman *et al.* (2009 ▶); Kwan *et al.* (2007 ▶); Carter *et al.* (2004 ▶); Wicker *et al.* (2007 ▶).
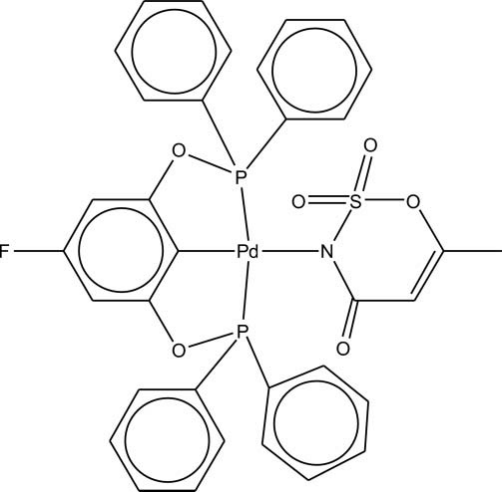


## Experimental

### 

#### Crystal data

[Pd(C_30_H_22_FO_2_P_2_)(C_4_H_4_NO_4_S)]
*M* *_r_* = 763.96
Triclinic, 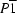

*a* = 10.6088 (12) Å
*b* = 11.0069 (7) Å
*c* = 14.066 (2) Å
α = 89.175 (9)°
β = 88.524 (12)°
γ = 82.021 (7)°
*V* = 1625.9 (3) Å^3^
*Z* = 2
Mo *K*α radiation
μ = 0.79 mm^−1^
*T* = 290 K
0.67 × 0.55 × 0.25 mm

#### Data collection

Enraf–Nonius CAD-4 diffractometer
Absorption correction: ψ scan (North *et al.*, 1968 ▶) *T*_min_ = 0.620, *T*_max_ = 0.828
6311 measured reflections
5963 independent reflections
5091 reflections with *I* > 2σ(*I*)
*R*_int_ = 0.020
3 standard reflections every 120 min  intensity decay: none

#### Refinement

*R*[*F* ^2^ > 2σ(*F* ^2^)] = 0.028
*wR*(*F* ^2^) = 0.078
*S* = 1.05
5963 reflections
416 parameters
H-atom parameters constrained
Δρ_max_ = 0.32 e Å^−3^
Δρ_min_ = −0.41 e Å^−3^

### 

Data collection: *CAD-4-PC* (Enraf–Nonius, 1993 ▶); cell refinement: *CAD-4-PC*; data reduction: *XCAD4-PC* (Harms & Wocadlo, 1995 ▶); program(s) used to solve structure: *SHELXS97* (Sheldrick, 2008 ▶); program(s) used to refine structure: *SHELXL97* (Sheldrick, 2008 ▶); molecular graphics: *OLEX2* (Dolomanov *et al.*, 2009 ▶); software used to prepare material for publication: *publCIF* (Westrip, 2010 ▶).

## Supplementary Material

Crystal structure: contains datablocks I, global. DOI: 10.1107/S1600536811002911/gw2097sup1.cif

Structure factors: contains datablocks I. DOI: 10.1107/S1600536811002911/gw2097Isup2.hkl

Additional supplementary materials:  crystallographic information; 3D view; checkCIF report

## Figures and Tables

**Table 1:** Hydrogen-bond geometry (Å, °)

*D*—H⋯*A*	*D*—H	H⋯*A*	*D*⋯*A*	*D*—H⋯*A*
C23—H23⋯O3^i^	0.93	2.42	3.284 (4)	155
C16—H16⋯O6^ii^	0.93	2.47	3.284 (4)	146
C10—H10⋯O4^iii^	0.93	2.50	3.305 (5)	145
C8—H8⋯F1^iv^	0.93	2.48	3.406 (5)	173

Symmetry codes: (i) 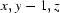; (ii) 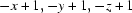; (iii) 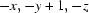; (iv) 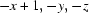.

**Fig. 1.**
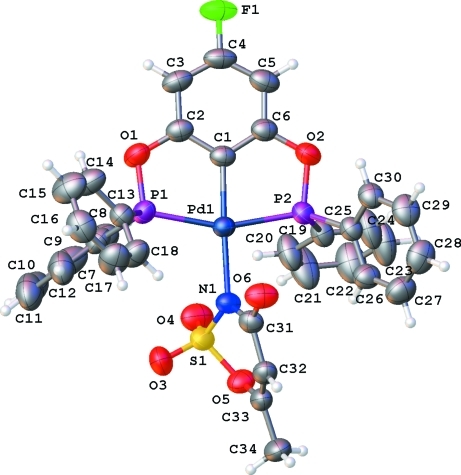
A thermal ellipsoid plot (50%) of the title compound showing the labeling scheme. H atoms are shown as spheres of arbitrary size.

## supplementary crystallographic information

### Comment

As thoroughly tested, apparently innocuous foodstuff additives (Lipinsky, 2003), the attraction of the acesulfamate and saccharinate anions as weakly binding ligands for organotransition-metal complexes and components of ionic liquids is obvious. The title complex, abbreviated as Ph{Pd_F_}Ace hereafter, is readily prepared in high yield by a benchtop procedure from its chlorido analogue (Kwan *et al.*, 2007; Hoffman *et al.*, 2009) in an extension of Rh(I) Vaska chemistry (Carter *et al.*, 2004). The presence of the pincer-ligand fluorine atom on the central aryl ring affords a convenient ^19^F-NMR reporter moiety (whose accuracy may also be corroborated by the two equivalent pendant-arm phosphinite donors by ^31^P-NMR) for monitoring ligand-substitution chemistry of the acesulfamate anion. A previous study of anion-metathesis equilibria using neutral Ph{Pd_F_}*X* and N(PPh_3_)_2_^+^ salts has shown anion affinity for Ph{Pd_F_}^+^ to follow *X*^-^ = chloride > saccharinate > trifluoroacetate > acesulfamate > nitrate (Wicker *et al.*, 2007). The acesulfamate complex displays a fascinating combination of ^19^F and ^31^P couplings to ^13^C nuclei in the pincer central fluoro-aryl ring, in which J_F—C_ and J_P—C_ correspond visually to their respective doublet and triplet coupling patterns.

A survey of crystal structures of transition-metal acesulfamate complexes shows three principal forms of metal–acesulfamate bonding: (i) monodentate metal to N bond, (ii) monodentate metal to carbonyl O bond (Şahin *et al.*, 2010; Dege *et al.*, 2007), and (iii) metal bonds to both N and carbonyl O in κ^2^-manner (Şahin *et al.*, 2009; Dege *et al.*, 2006; Bulut *et al.*, 2005). When water is present in the crystal, extensive hydrogen bonding occurs. The title compound, a four-coordinate d^8^ complex, has the expected distorted square-planar geometry in which the acesulfamate is N-bound to Pd. The Pd—N distance is 2.127 (2) Å, significantly longer than the Pt—N distance (2.036 (3) Å) in another square-planar d^8^ complex, K_2_[*trans*-Pt(Ace)_2_Cl_2_] (Cavicchioli *et al.*, 2010; Beck *et al.*, 1985), but much shorter than those in divalent late-metal N-acesulfamates with octahedral Jahn-Teller distortion: 2.7175 (16) Å in *trans*-Cu(c—C_6_H_10_-1,2-(NH_2_)_2_)(Ace)_2_ (Şahin *et al.*, 2010) and 2.3180 (19) Å in *trans*-Co(H_2_O)_4_(Ace)_2_ (İçbudak, 2005). The only directly comparable structure above, K_2_[*trans*-Pt(Ace)_2_Cl_2_], probably has a shorter Pt—N distance than that in Ph{Pd_F_}Ace because the *trans*-effect of the fluoroaryl-Pd moiety is stronger than that of N-bound acesulfamate. The almost-planar acesulfamate ring (with the sulfur the only significantly nonplanar atom) in Ph{Pd_F_}Ace is tilted 76.35 (6)° from the Pd(II) coordination plane, whereas the similarly nearly-planar acesulfamate ring in K_2_[*trans*-Pt(Ace)_2_Cl_2_] is nearly perpendicular to the Pt(II) coordination plane.

### Experimental

Pd(C_4_H_4_NO_4_S)(C_18_H_22_FO_2_P_2_), abbreviated by Ph{Pd_F_}Ace hereafter, was prepared by stirring Ph{Pd_F_}Cl (25 mg, 0.040 mmol) with silver(I) acesulfamate (1.5 mol equiv.) in benzene (25 mL) at ambient temperature for 24 h. The AgCl precipitate was then removed by filtration, and the solvent was removed on a rotary evapaorator to afford a white microcrystalline solid (88% yield). Suitable single crystals were prepared by slow evaporation of solvent from a solution in fluorobenzene at 24 °C. The complex was characterized by NMR in CDCl_3_.

^1^H: (relative to internal TMS) δ 1.999 and δ 2.015 (3*H*, apparent doublet); δ 5.476 and δ 5.480 (1*H*, apparent doublet); δ 6.443 (2*H*, doublet, ^3^J_H—F_=9.7 Hz); δ 7.51 (8*H*, overlapping multiplets); δ 7.807 (12*H*, overlapping multiplets)

^19^F: (relative to internal C_6_F_6_ at δ -161.59) δ -111.29 (triplet of triplets,^3^J_H—F_=9.7 Hz, ^5^J_P—F_=1.9 Hz)

^31^P: (relative to external 85% aq. phosphoric acid) δ 148.79 (doublet, ^5^J_P—F_=1.9 Hz)

^13^C: (relative to internal TMS) Ace: δ 19.47(*s*), δ 103.34(*s*), carbonyl not observed with 6 K scans Ph_2_P: δ 128.69(t; ^3^J_P—C_=5.8 Hz), ? 132.35(*s*), δ 132.43(t; ^1^J_P—C_=53 Hz), δ 132.54(t; ^2^J_P—C_=8.2 Hz) Pincer Aryl: δ 95.69(d of t; ^2^J_F—C_=26 Hz), ^3^J_P—C_=8.6 Hz), δ 123.68(d of t; ^3^J_F—C_~^2^J_P—C_\sim 32 Hz, ^3^J_P—C_=8.6 Hz), δ 164.07(d of t; ^3^J_F—C_=15 Hz, ^2^J_P—C_=7.7 Hz), δ 163.93(d; ^1^J_F—C_=244 Hz)

### Refinement

Hydrogen atoms were placed in calculated positions and allowed to ride during subsequent refinement, with *U*_iso_(H) = 1.2*U*_eq_(C) and C—H distances of 0.93 Å for all H atoms except for the methyl H atoms which were refined with *U*_iso_(H) = 1.5*U*_eq_(C) and C—H distances of 0.96 Å.

### Crystal data

**Table:**

[Pd(C_30_H_22_FO_2_P_2_)(C_4_H_4_NO_4_S)]	*Z* = 2
*M**_r_* = 763.96	*F*(000) = 772
Triclinic, *P*1	*D*_x_ = 1.560 Mg m^−^^3^
Hall symbol: -P 1	Mo *K*α radiation, λ = 0.71073 Å
*a* = 10.6088 (12) Å	Cell parameters from 25 reflections
*b* = 11.0069 (7) Å	θ = 8.0–12.0°
*c* = 14.066 (2) Å	µ = 0.79 mm^−^^1^
α = 89.175 (9)°	*T* = 290 K
β = 88.524 (12)°	Prism, colorless
γ = 82.021 (7)°	0.67 × 0.55 × 0.25 mm
*V* = 1625.9 (3) Å^3^	

### Data collection

**Table:**

Enraf–Nonius CAD-4 diffractometer	5091 reflections with *I* > 2σ(*I*)
Radiation source: fine-focus sealed tube	*R*_int_ = 0.020
graphite	θ_max_ = 25.4°, θ_min_ = 2.4°
θ/2θ scans	*h* = 0→12
Absorption correction: ψ scan (North *et al.*, 1968)	*k* = −13→13
*T*_min_ = 0.620, *T*_max_ = 0.828	*l* = −16→16
6311 measured reflections	3 standard reflections every 120 min
5963 independent reflections	

### Refinement

**Table:**

Refinement on *F*^2^	Primary atom site location: structure-invariant direct methods
Least-squares matrix: full	Secondary atom site location: difference Fourier map
*R*[*F*^2^ > 2σ(*F*^2^)] = 0.028	Hydrogen site location: inferred from neighbouring sites
*wR*(*F*^2^) = 0.078	H-atom parameters constrained
*S* = 1.05	*w* = 1/[σ^2^(*F*_o_^2^) + (0.0404*P*)^2^ + 0.4794*P*] where *P* = (*F*_o_^2^ + 2*F*_c_^2^)/3
5963 reflections	(Δ/σ)_max_ = 0.001
416 parameters	Δρ_max_ = 0.32 e Å^−^^3^
0 restraints	Δρ_min_ = −0.41 e Å^−^^3^

### Special details

**Table:**

Geometry. All e.s.d.'s (except the e.s.d. in the dihedral angle between two l.s. planes) are estimated using the full covariance matrix. The cell e.s.d.'s are taken into account individually in the estimation of e.s.d.'s in distances, angles and torsion angles; correlations between e.s.d.'s in cell parameters are only used when they are defined by crystal symmetry. An approximate (isotropic) treatment of cell e.s.d.'s is used for estimating e.s.d.'s involving l.s. planes.
Refinement. Refinement of *F*^2^ against ALL reflections. The weighted *R*-factor *wR* and goodness of fit *S* are based on *F*^2^, conventional *R*-factors *R* are based on *F*, with *F* set to zero for negative *F*^2^. The threshold expression of *F*^2^ > 2σ(*F*^2^) is used only for calculating *R*-factors(gt) *etc*. and is not relevant to the choice of reflections for refinement. *R*-factors based on *F*^2^ are statistically about twice as large as those based on *F*, and *R*- factors based on ALL data will be even larger.

### Fractional atomic coordinates and isotropic or equivalent isotropic displacement parameters (Å^2^)

**Table:**

	*x*	*y*	*z*	*U*_iso_*/*U*_eq_	
Pd1	0.319883 (17)	0.194802 (18)	0.257293 (13)	0.03998 (7)	
P1	0.40296 (7)	0.34887 (7)	0.17797 (5)	0.04728 (17)	
P2	0.29164 (7)	0.00104 (6)	0.30445 (5)	0.04611 (16)	
S1	0.04728 (7)	0.36685 (7)	0.28910 (5)	0.05509 (19)	
F1	0.73387 (18)	−0.1038 (2)	0.01746 (14)	0.0793 (6)	
O1	0.5064 (2)	0.27851 (19)	0.10175 (14)	0.0594 (5)	
O2	0.4125 (2)	−0.08849 (18)	0.25628 (15)	0.0601 (5)	
O3	0.0472 (3)	0.4957 (2)	0.29368 (18)	0.0809 (7)	
O4	0.0277 (2)	0.3168 (2)	0.20004 (16)	0.0760 (7)	
O5	−0.07029 (18)	0.3374 (2)	0.35586 (15)	0.0599 (5)	
O6	0.2777 (2)	0.2668 (2)	0.47308 (15)	0.0652 (6)	
N1	0.1673 (2)	0.2921 (2)	0.33821 (16)	0.0480 (5)	
C1	0.4573 (2)	0.0973 (2)	0.18193 (18)	0.0445 (6)	
C2	0.5307 (3)	0.1527 (3)	0.11519 (19)	0.0492 (6)	
C3	0.6248 (3)	0.0868 (3)	0.0595 (2)	0.0562 (7)	
H3	0.6732	0.1253	0.0154	0.067*	
C4	0.6432 (3)	−0.0378 (3)	0.0727 (2)	0.0590 (8)	
C5	0.5753 (3)	−0.1000 (3)	0.1369 (2)	0.0584 (8)	
H5	0.5905	−0.1849	0.1436	0.070*	
C6	0.4836 (3)	−0.0294 (3)	0.19080 (19)	0.0497 (6)	
C7	0.3064 (3)	0.4549 (3)	0.1022 (2)	0.0555 (7)	
C8	0.2366 (3)	0.4084 (4)	0.0329 (2)	0.0768 (10)	
H8	0.2418	0.3241	0.0246	0.092*	
C9	0.1581 (4)	0.4901 (6)	−0.0243 (3)	0.0938 (14)	
H9	0.1132	0.4599	−0.0724	0.113*	
C10	0.1466 (4)	0.6124 (6)	−0.0106 (3)	0.1002 (16)	
H10	0.0940	0.6656	−0.0492	0.120*	
C11	0.2112 (4)	0.6571 (4)	0.0587 (3)	0.0974 (14)	
H11	0.2008	0.7413	0.0689	0.117*	
C12	0.2926 (4)	0.5806 (3)	0.1151 (3)	0.0772 (10)	
H12	0.3383	0.6132	0.1618	0.093*	
C13	0.4943 (3)	0.4430 (2)	0.2440 (2)	0.0485 (6)	
C14	0.6042 (3)	0.4790 (3)	0.2059 (3)	0.0725 (10)	
H14	0.6361	0.4494	0.1472	0.087*	
C15	0.6669 (4)	0.5593 (4)	0.2551 (3)	0.0830 (11)	
H15	0.7421	0.5823	0.2299	0.100*	
C16	0.6199 (4)	0.6049 (3)	0.3398 (3)	0.0718 (9)	
H16	0.6613	0.6609	0.3715	0.086*	
C17	0.5120 (4)	0.5686 (4)	0.3782 (3)	0.0786 (10)	
H17	0.4802	0.5996	0.4365	0.094*	
C18	0.4494 (3)	0.4861 (3)	0.3315 (2)	0.0668 (8)	
H18	0.3772	0.4598	0.3591	0.080*	
C19	0.1541 (3)	−0.0538 (3)	0.2582 (2)	0.0522 (7)	
C20	0.0796 (4)	0.0157 (3)	0.1943 (3)	0.0766 (10)	
H20	0.0995	0.0923	0.1757	0.092*	
C21	−0.0252 (4)	−0.0270 (4)	0.1573 (3)	0.1013 (15)	
H21	−0.0757	0.0213	0.1142	0.122*	
C22	−0.0549 (4)	−0.1389 (4)	0.1833 (3)	0.0922 (13)	
H22	−0.1257	−0.1672	0.1584	0.111*	
C23	0.0189 (5)	−0.2090 (4)	0.2457 (3)	0.0961 (14)	
H23	−0.0008	−0.2861	0.2632	0.115*	
C24	0.1219 (4)	−0.1674 (3)	0.2831 (3)	0.0805 (11)	
H24	0.1715	−0.2165	0.3262	0.097*	
C25	0.2974 (3)	−0.0480 (2)	0.42676 (19)	0.0458 (6)	
C26	0.1920 (3)	−0.0163 (3)	0.4854 (2)	0.0591 (7)	
H26	0.1176	0.0246	0.4602	0.071*	
C27	0.1961 (3)	−0.0446 (3)	0.5806 (2)	0.0718 (9)	
H27	0.1251	−0.0216	0.6198	0.086*	
C28	0.3057 (4)	−0.1074 (4)	0.6181 (2)	0.0733 (10)	
H28	0.3082	−0.1277	0.6825	0.088*	
C29	0.4097 (3)	−0.1394 (3)	0.5611 (2)	0.0665 (9)	
H29	0.4833	−0.1818	0.5867	0.080*	
C30	0.4077 (3)	−0.1095 (3)	0.4651 (2)	0.0543 (7)	
H30	0.4798	−0.1306	0.4267	0.065*	
C31	0.1763 (3)	0.2983 (2)	0.43552 (19)	0.0473 (6)	
C32	0.0581 (3)	0.3353 (2)	0.4901 (2)	0.0507 (6)	
H32	0.0638	0.3495	0.5548	0.061*	
C33	−0.0556 (3)	0.3498 (2)	0.4529 (2)	0.0508 (7)	
C34	−0.1806 (3)	0.3777 (3)	0.5027 (3)	0.0680 (9)	
H34A	−0.1680	0.3898	0.5690	0.102*	
H34B	−0.2283	0.3104	0.4955	0.102*	
H34C	−0.2268	0.4508	0.4759	0.102*	

### Atomic displacement parameters (Å^2^)

**Table:**

	*U*^11^	*U*^22^	*U*^33^	*U*^12^	*U*^13^	*U*^23^
Pd1	0.03722 (11)	0.04267 (12)	0.03896 (12)	−0.00192 (8)	0.00034 (8)	−0.00073 (8)
P1	0.0490 (4)	0.0496 (4)	0.0438 (4)	−0.0100 (3)	0.0060 (3)	−0.0018 (3)
P2	0.0459 (4)	0.0421 (4)	0.0485 (4)	0.0003 (3)	−0.0018 (3)	0.0037 (3)
S1	0.0510 (4)	0.0561 (4)	0.0535 (4)	0.0074 (3)	0.0034 (3)	0.0106 (3)
F1	0.0624 (11)	0.0990 (15)	0.0679 (12)	0.0194 (10)	0.0124 (9)	−0.0230 (11)
O1	0.0628 (12)	0.0598 (12)	0.0561 (12)	−0.0139 (10)	0.0193 (10)	−0.0079 (10)
O2	0.0623 (12)	0.0488 (11)	0.0633 (13)	0.0112 (9)	0.0086 (10)	0.0036 (10)
O3	0.0935 (18)	0.0538 (13)	0.0895 (18)	0.0051 (12)	0.0168 (14)	0.0220 (12)
O4	0.0704 (15)	0.0988 (18)	0.0523 (13)	0.0119 (13)	−0.0092 (11)	0.0079 (12)
O5	0.0412 (10)	0.0740 (14)	0.0623 (13)	−0.0011 (9)	0.0014 (9)	0.0057 (11)
O6	0.0520 (12)	0.0866 (16)	0.0544 (12)	0.0023 (11)	−0.0091 (10)	−0.0136 (11)
N1	0.0442 (12)	0.0517 (13)	0.0448 (12)	0.0043 (10)	0.0022 (10)	−0.0004 (10)
C1	0.0389 (13)	0.0518 (15)	0.0410 (13)	0.0010 (11)	−0.0035 (11)	−0.0053 (11)
C2	0.0437 (14)	0.0609 (17)	0.0432 (14)	−0.0064 (12)	−0.0008 (11)	−0.0105 (13)
C3	0.0425 (15)	0.080 (2)	0.0460 (15)	−0.0083 (14)	0.0048 (12)	−0.0128 (14)
C4	0.0452 (15)	0.079 (2)	0.0478 (16)	0.0112 (14)	−0.0015 (13)	−0.0181 (15)
C5	0.0551 (17)	0.0610 (18)	0.0542 (17)	0.0122 (14)	−0.0091 (14)	−0.0082 (14)
C6	0.0439 (14)	0.0570 (17)	0.0457 (15)	0.0029 (12)	−0.0043 (12)	−0.0018 (13)
C7	0.0491 (16)	0.073 (2)	0.0462 (16)	−0.0160 (14)	0.0047 (12)	0.0090 (14)
C8	0.070 (2)	0.106 (3)	0.055 (2)	−0.015 (2)	0.0013 (17)	−0.0060 (19)
C9	0.062 (2)	0.169 (5)	0.050 (2)	−0.014 (3)	−0.0069 (17)	0.000 (3)
C10	0.071 (3)	0.146 (5)	0.077 (3)	0.002 (3)	0.001 (2)	0.045 (3)
C11	0.087 (3)	0.091 (3)	0.113 (4)	−0.013 (2)	−0.016 (3)	0.046 (3)
C12	0.076 (2)	0.067 (2)	0.089 (3)	−0.0160 (18)	−0.015 (2)	0.0237 (19)
C13	0.0485 (15)	0.0468 (15)	0.0496 (15)	−0.0048 (12)	−0.0025 (12)	0.0000 (12)
C14	0.0609 (19)	0.092 (3)	0.069 (2)	−0.0257 (18)	0.0137 (16)	−0.0235 (19)
C15	0.069 (2)	0.099 (3)	0.088 (3)	−0.037 (2)	0.006 (2)	−0.021 (2)
C16	0.079 (2)	0.068 (2)	0.072 (2)	−0.0172 (18)	−0.0230 (19)	−0.0061 (17)
C17	0.101 (3)	0.080 (2)	0.058 (2)	−0.023 (2)	0.0042 (19)	−0.0165 (18)
C18	0.072 (2)	0.073 (2)	0.0579 (19)	−0.0200 (17)	0.0114 (16)	−0.0104 (16)
C19	0.0602 (17)	0.0455 (15)	0.0512 (16)	−0.0065 (13)	−0.0111 (13)	0.0035 (12)
C20	0.082 (2)	0.0583 (19)	0.093 (3)	−0.0176 (17)	−0.037 (2)	0.0232 (18)
C21	0.099 (3)	0.082 (3)	0.129 (4)	−0.025 (2)	−0.068 (3)	0.034 (3)
C22	0.094 (3)	0.083 (3)	0.108 (3)	−0.034 (2)	−0.045 (3)	0.013 (2)
C23	0.134 (4)	0.066 (2)	0.099 (3)	−0.047 (2)	−0.046 (3)	0.021 (2)
C24	0.110 (3)	0.0525 (18)	0.084 (2)	−0.0237 (19)	−0.043 (2)	0.0206 (17)
C25	0.0463 (14)	0.0422 (14)	0.0488 (15)	−0.0048 (11)	−0.0070 (12)	0.0013 (11)
C26	0.0516 (17)	0.0661 (19)	0.0572 (18)	0.0002 (14)	−0.0026 (14)	0.0023 (15)
C27	0.070 (2)	0.089 (3)	0.0562 (19)	−0.0098 (18)	0.0051 (16)	−0.0014 (17)
C28	0.080 (2)	0.092 (3)	0.0510 (18)	−0.023 (2)	−0.0165 (17)	0.0097 (17)
C29	0.0622 (19)	0.072 (2)	0.067 (2)	−0.0127 (16)	−0.0270 (17)	0.0109 (17)
C30	0.0464 (15)	0.0555 (16)	0.0611 (18)	−0.0056 (13)	−0.0092 (13)	0.0043 (14)
C31	0.0492 (15)	0.0430 (14)	0.0492 (15)	−0.0042 (12)	0.0003 (12)	−0.0079 (12)
C32	0.0571 (17)	0.0472 (15)	0.0470 (15)	−0.0056 (12)	0.0076 (13)	−0.0053 (12)
C33	0.0521 (16)	0.0416 (14)	0.0572 (17)	−0.0041 (12)	0.0105 (13)	0.0028 (12)
C34	0.0542 (18)	0.065 (2)	0.082 (2)	−0.0041 (15)	0.0226 (16)	0.0038 (17)

### Geometric parameters (Å, °)

**Table:**

Pd1—C1	1.979 (3)	C13—C14	1.376 (4)
Pd1—N1	2.127 (2)	C14—C15	1.381 (5)
Pd1—P2	2.2814 (7)	C14—H14	0.9300
Pd1—P1	2.2819 (8)	C15—C16	1.355 (5)
P1—O1	1.637 (2)	C15—H15	0.9300
P1—C13	1.797 (3)	C16—C17	1.361 (5)
P1—C7	1.799 (3)	C16—H16	0.9300
P2—O2	1.642 (2)	C17—C18	1.379 (5)
P2—C19	1.795 (3)	C17—H17	0.9300
P2—C25	1.796 (3)	C18—H18	0.9300
S1—O4	1.408 (2)	C19—C20	1.366 (4)
S1—O3	1.420 (2)	C19—C24	1.378 (4)
S1—N1	1.585 (2)	C20—C21	1.382 (5)
S1—O5	1.608 (2)	C20—H20	0.9300
F1—C4	1.357 (3)	C21—C22	1.356 (5)
O1—C2	1.384 (3)	C21—H21	0.9300
O2—C6	1.387 (3)	C22—C23	1.351 (5)
O5—C33	1.389 (4)	C22—H22	0.9300
O6—C31	1.215 (3)	C23—C24	1.363 (5)
N1—C31	1.378 (3)	C23—H23	0.9300
C1—C6	1.389 (4)	C24—H24	0.9300
C1—C2	1.392 (4)	C25—C26	1.380 (4)
C2—C3	1.382 (4)	C25—C30	1.386 (4)
C3—C4	1.369 (5)	C26—C27	1.370 (4)
C3—H3	0.9300	C26—H26	0.9300
C4—C5	1.375 (5)	C27—C28	1.381 (5)
C5—C6	1.376 (4)	C27—H27	0.9300
C5—H5	0.9300	C28—C29	1.357 (5)
C7—C8	1.384 (5)	C28—H28	0.9300
C7—C12	1.385 (5)	C29—C30	1.384 (4)
C8—C9	1.399 (6)	C29—H29	0.9300
C8—H8	0.9300	C30—H30	0.9300
C9—C10	1.350 (7)	C31—C32	1.465 (4)
C9—H9	0.9300	C32—C33	1.316 (4)
C10—C11	1.343 (6)	C32—H32	0.9300
C10—H10	0.9300	C33—C34	1.479 (4)
C11—C12	1.378 (5)	C34—H34A	0.9600
C11—H11	0.9300	C34—H34B	0.9600
C12—H12	0.9300	C34—H34C	0.9600
C13—C18	1.375 (4)		
			
C1—Pd1—N1	177.21 (10)	C14—C13—P1	121.0 (2)
C1—Pd1—P2	79.58 (8)	C13—C14—C15	119.8 (3)
N1—Pd1—P2	97.94 (7)	C13—C14—H14	120.1
C1—Pd1—P1	79.99 (8)	C15—C14—H14	120.1
N1—Pd1—P1	102.47 (7)	C16—C15—C14	120.6 (3)
P2—Pd1—P1	159.56 (3)	C16—C15—H15	119.7
O1—P1—C13	103.92 (12)	C14—C15—H15	119.7
O1—P1—C7	101.87 (13)	C15—C16—C17	119.8 (3)
C13—P1—C7	104.64 (14)	C15—C16—H16	120.1
O1—P1—Pd1	104.63 (8)	C17—C16—H16	120.1
C13—P1—Pd1	118.05 (10)	C16—C17—C18	120.6 (3)
C7—P1—Pd1	121.20 (10)	C16—C17—H17	119.7
O2—P2—C19	104.21 (13)	C18—C17—H17	119.7
O2—P2—C25	102.21 (12)	C13—C18—C17	119.8 (3)
C19—P2—C25	105.35 (13)	C13—C18—H18	120.1
O2—P2—Pd1	104.98 (8)	C17—C18—H18	120.1
C19—P2—Pd1	115.23 (9)	C20—C19—C24	117.9 (3)
C25—P2—Pd1	122.61 (9)	C20—C19—P2	120.2 (2)
O4—S1—O3	117.70 (16)	C24—C19—P2	121.9 (2)
O4—S1—N1	110.61 (13)	C19—C20—C21	120.4 (3)
O3—S1—N1	112.39 (15)	C19—C20—H20	119.8
O4—S1—O5	105.38 (14)	C21—C20—H20	119.8
O3—S1—O5	105.92 (13)	C22—C21—C20	120.4 (3)
N1—S1—O5	103.46 (12)	C22—C21—H21	119.8
C2—O1—P1	114.26 (17)	C20—C21—H21	119.8
C6—O2—P2	113.99 (17)	C23—C22—C21	119.6 (4)
C33—O5—S1	115.71 (18)	C23—C22—H22	120.2
C31—N1—S1	118.54 (18)	C21—C22—H22	120.2
C31—N1—Pd1	119.49 (17)	C22—C23—C24	120.4 (3)
S1—N1—Pd1	121.80 (13)	C22—C23—H23	119.8
C6—C1—C2	116.5 (2)	C24—C23—H23	119.8
C6—C1—Pd1	122.2 (2)	C23—C24—C19	121.3 (3)
C2—C1—Pd1	121.3 (2)	C23—C24—H24	119.4
C3—C2—O1	118.3 (3)	C19—C24—H24	119.4
C3—C2—C1	122.7 (3)	C26—C25—C30	119.2 (3)
O1—C2—C1	118.9 (2)	C26—C25—P2	119.1 (2)
C4—C3—C2	116.6 (3)	C30—C25—P2	121.6 (2)
C4—C3—H3	121.7	C27—C26—C25	120.7 (3)
C2—C3—H3	121.7	C27—C26—H26	119.7
F1—C4—C3	117.3 (3)	C25—C26—H26	119.7
F1—C4—C5	118.2 (3)	C26—C27—C28	119.9 (3)
C3—C4—C5	124.5 (3)	C26—C27—H27	120.1
C4—C5—C6	116.2 (3)	C28—C27—H27	120.1
C4—C5—H5	121.9	C29—C28—C27	120.0 (3)
C6—C5—H5	121.9	C29—C28—H28	120.0
C5—C6—O2	118.1 (3)	C27—C28—H28	120.0
C5—C6—C1	123.4 (3)	C28—C29—C30	120.7 (3)
O2—C6—C1	118.4 (2)	C28—C29—H29	119.6
C8—C7—C12	119.0 (3)	C30—C29—H29	119.6
C8—C7—P1	118.6 (3)	C29—C30—C25	119.6 (3)
C12—C7—P1	122.3 (3)	C29—C30—H30	120.2
C7—C8—C9	118.9 (4)	C25—C30—H30	120.2
C7—C8—H8	120.5	O6—C31—N1	120.1 (2)
C9—C8—H8	120.5	O6—C31—C32	122.6 (3)
C10—C9—C8	121.0 (4)	N1—C31—C32	117.1 (2)
C10—C9—H9	119.5	C33—C32—C31	123.8 (3)
C8—C9—H9	119.5	C33—C32—H32	118.1
C11—C10—C9	120.0 (4)	C31—C32—H32	118.1
C11—C10—H10	120.0	C32—C33—O5	121.0 (2)
C9—C10—H10	120.0	C32—C33—C34	128.0 (3)
C10—C11—C12	121.2 (5)	O5—C33—C34	111.0 (3)
C10—C11—H11	119.4	C33—C34—H34A	109.5
C12—C11—H11	119.4	C33—C34—H34B	109.5
C11—C12—C7	119.8 (4)	H34A—C34—H34B	109.5
C11—C12—H12	120.1	C33—C34—H34C	109.5
C7—C12—H12	120.1	H34A—C34—H34C	109.5
C18—C13—C14	119.4 (3)	H34B—C34—H34C	109.5
C18—C13—P1	119.4 (2)		

### Hydrogen-bond geometry (Å, °)

**Table:**

*D*—H···*A*	*D*—H	H···*A*	*D*···*A*	*D*—H···*A*
C23—H23···O3^i^	0.93	2.42	3.284 (4)	155
C16—H16···O6^ii^	0.93	2.47	3.284 (4)	146
C10—H10···O4^iii^	0.93	2.50	3.305 (5)	145
C8—H8···F1^iv^	0.93	2.48	3.406 (5)	173

Symmetry codes: (i) *x*, *y*−1, *z*; (ii) −*x*+1, −*y*+1, −*z*+1; (iii) −*x*, −*y*+1, −*z*; (iv) −*x*+1, −*y*, −*z*.
